# Clinical efficacy and safety of percutaneous coronary intervention for acute myocardial infarction complicated with chronic renal insufficiency: A protocol of systematic review and meta-analysis

**DOI:** 10.1097/MD.0000000000016005

**Published:** 2019-06-14

**Authors:** Liang Luo, Wen-Qing Xu, Ri-Xiang Zhong, Feng Chen, You-Lin Fu, Peng Zhang, Shi-Hui Xiao

**Affiliations:** a NO.2 Department of Internal Medicine-Cardiovascular, Ganzhou People's Hospital; b NO.1 Department of Internal Medicine, First People's Hospital of Longnan County; c NO.1 Department of Internal Medicine-Cardiovascular, Ganzhou People's Hospital, Ganzhou, Jiangxi Province, 341000, China.

**Keywords:** acute myocardial infarction, chronic renal insufficiency, efficacy and safety, meta-analysis, percutaneous coronary intervention, randomized controlled trials

## Abstract

**Background:**

The aim of this research is to further evaluate the efficacy and safety of percutaneous coronary intervention (PCI) in patients with acute myocardial infarction (AMI) complicated with chronic renal insufficiency (CRI) by meta-analysis, to provide scientific and effective medical evidence for PCI in patients with AMI complicated with CRI, and to support the clinical application of PCI.

**Methods:**

Electronic databases will be searched, including PubMed, Cochrane Library, Embase, CNKI, CBM, VIP, and Wanfang Data. Patients with AMI complicated by renal insufficiency treated with PCI will be included. The retrieval time is from inception to January 2019. The inclusion and exclusion criteria are formulated to search only the relevant literature. Endnote software management for literature will be adopted. The literature will be independently screened by 2 researchers. Excel 2016 will be applied to extract literature data with the “Research Information Registration Form.” The final selected literature will be assessed for bias risk. Stata 12.0 software will be used for the meta-analysis.

**Results:**

The systematic evaluation and meta-analysis will be carried out strictly in accordance with the requirements of the Cochrane System Evaluator Manual 5.3 on meta-analyses, which will provide a high-quality evaluation of the clinical efficacy and safety of PCI in patients with AMI and CRI.

**Ethics and dissemination:**

This study belongs to the category of systematic reviews, not clinical trials. Therefore, it does not require ethical approval. The results of this study will be published in influential international academic journals related to this topic.

**Conclusion:**

PCI is an effective and safe treatment for patients with AMI and CRI. This study will provide a definite evidence-based medical conclusion and provide a scientific basis for the clinical treatment of patients with AMI and CRI.

**PROSPERO registration number:**

CRD42019131367.

## 1 Introduction

Myocardial infarction (MI) is a common term^[1]^ for myocardial ischemic necrosis or global MI. Patients usually suffer coronary artery atherosclerosis and stenosis. Coronary atherosclerotic plaque occurs because of some inducements (increased aerobic during activity, among othdrs). The platelet components in the blood aggregate on the surface of the ruptured plaque and form a thrombus. Then, the coronary artery lumen is blocked, resulting in insufficient blood supply to the myocardium and ischemic necrosis of the myocardium.^[2,3]^ Coronary artery spasm may also induce acute myocardial infarction (AMI).^[4,5]^ AMI is a common acute and severe cardiovascular disease in the clinic. The incidence of AMI in China is positively correlated with the increase in the aging population, the fast pace of life, the change in dietary habits, the influence of complex social and psychological factors, among others.^[5,6]^ Therefore, effective treatment and prevention of MI have become a focus of current cardiac research.

Chronic renal insufficiency (CRI) is a common comorbidity of AMI. In recent years, many studies have shown that CRI is an independent risk factor for poor prognosis in patients with AMI.^[7]^ Some scholars at home and abroad believe that the decline in or damage to cardiac function caused by CRI is closely related to the accumulation of endogenous creatinine, metabolites, and other toxic products in vivo. However, many other factors may lead to damage to cardiac function and myocardial cells.^[8]^ Clinically, there are many complications of chronic renal failure with MI. Additionally, they cover a wide range, and the main pathological mechanism is still unclear. The possible causes are as follows: excessive oxidative stress, renin-angiotensin-aldosterone system activation, microvascular inflammation, lipid metabolism disorder, and the nonmetabolic toxins in end-stage renal disease, which can increase to different degrees in patients with CRI.^[9,10]^ Percutaneous coronary intervention (PCI) refers to the method of improving myocardial perfusion therapy by dredging a narrow or even occluded coronary artery lumen through cardiac catheterization. Coronary artery intervention technology belongs to the category of vascular recanalization and is the least traumatic method of myocardial blood flow reconstruction.^[11]^ Previous studies have shown that renal insufficiency increases the poor prognosis of cardiovascular diseases.^[12]^ Recent reports have shown that renal impairment is common in patients with acute coronary syndrome and is associated with a high risk of death, even when perfusing with the most effective method, such as PCI.^[13,14]^ Owing to the lack of evidence-based medical evidence about PCI in the treatment of AMI with CRI, the purpose of this study is to evaluate the safety, effectiveness, and long-term prognosis of PCI in the treatment of AMI with CRI and to provide evidence-based medical evidence for clinical treatment.

## 2 Methods and analysis

### 2.1 PROSPERO registration

This systematic evaluation has been registered on the PROSPERO International prospective register of systematic reviews website (https://www.crd.york.ac.uk/PROSPERO/), and the registration number is CRD42019131367.

### 2.2 Study inclusion and exclusion criteria

#### 2.2.1 Types of studies

##### 2.2.1.1 Eligibility criteria

The selected literature must be a clinical study of percutaneous coronary intervention in the treatment of acute myocardial infarction complicated with chronic renal insufficiency. There are no restrictions on the language of the selection study, nor whether blinding or assigning hidden restrictions are used in the research process.

#### 2.2.2 Types of participants

##### 2.2.2.1 Inclusion criteria

The inclusion criteria are:

1. The studies are observational studies of cases and controls.
2. The publication language is not restricted.
3. The subjects are patients with AMI complicated with renal insufficiency treated by PCI.
4. Patients meet the diagnostic criteria of AMI,^[15]^ also called STEMI, namely, ≥2 of the following 3 items:
  - History of ischemic chest pain: AMI pain is usually transduced to the left upper arm, jaw, back or shoulder, usually behind the sternum or left chest; sometimes the pain is atypical and can be located in the upper abdomen, neck, mandible and other parts. Pain often lasts for >20 minutes, usually with severe pressure, urgency, or a burning sensation, often accompanied by dyspnea, sweating, nausea, vomiting, vertigo, among others.
  - Electrocardiographic changes: there are >2 related leads with the ST segment rising upward and/or showing dynamic changes at the ST segment.
  - Serum enzymatic changes: serum myocardial necrosis markers, especially cardiac troponin I (cTnI), are increased.
5. Patients meet the diagnostic criteria of CRI:^[16]^ GFR (mL/min 1.73 m^2^) = 186 × (Scr) – 1.154 × (age) – 0.203 × (0.742 female), wherein GFR is glomerular filtration rate, Scr is serum creatinine (mg/dL), age is calculated in years, and weight is calculated in kilograms.

##### 2.2.2.2 Exclusion criteria

Documents that meet the following criteria will be excluded: abstracts without full text; no control group; incomplete descriptions of outcome indicators; without statistical analysis; reviews or animal experiments; repeat publication of research results.^[17]^

#### 2.2.3 Types of interventions.

##### 2.2.3.1 Experimental group

The experimental group was patients with AMI combined with CRI who underwent PCI.

##### 2.2.3.2 Control group

Control group comprises patients with AMI and normal renal function who underwent PCI.

### 2.3 Types of outcome measures

#### 2.3.1 Main outcome indicators

Main outcome indicators are coronary lesions; and incidence of major adverse cardiovascular events

#### 2.3.2 Secondary outcomes

Secondary outcomes are long-term mortality, recovery after operation, and length of hospital stay.

### 2.4 Search strategy

#### 2.4.1 Electronic searches

The retrieval strategy is to search the electronic database of the network by computer, including PubMed, Cochrane Library, Embase, CNKI, CBM, VIP, and Wanfang database, to collect the literature on PCI treatment for elderly patients with AMI complicated with renal insufficiency. The retrieval time will be from inception to January 2019. All database searches will be based on the combination of subject words and free words and will be adjusted according to the specific database. Retrieval strategies will be determined by multiple researches. Chinese search terms will include “Ji-xing-xin-ji-geng-si,” “Man-xing-shen-gong-neng-bu-quan,” “Man-xing-shen-shuai-jie,” and “Jing-pi-guan-zhuang-dong-mai-jie-ru-zhi-liao,” English search terms will include “AMI,” “CKD,” “Acute myocardial,” “Chronic renal failure,” and “Percutaneous coronary intervention.” The retrieval strategy of 3 databases, that is, PubMed, Embase, and SinoMed, is shown in Table 1.

**Table 1:**
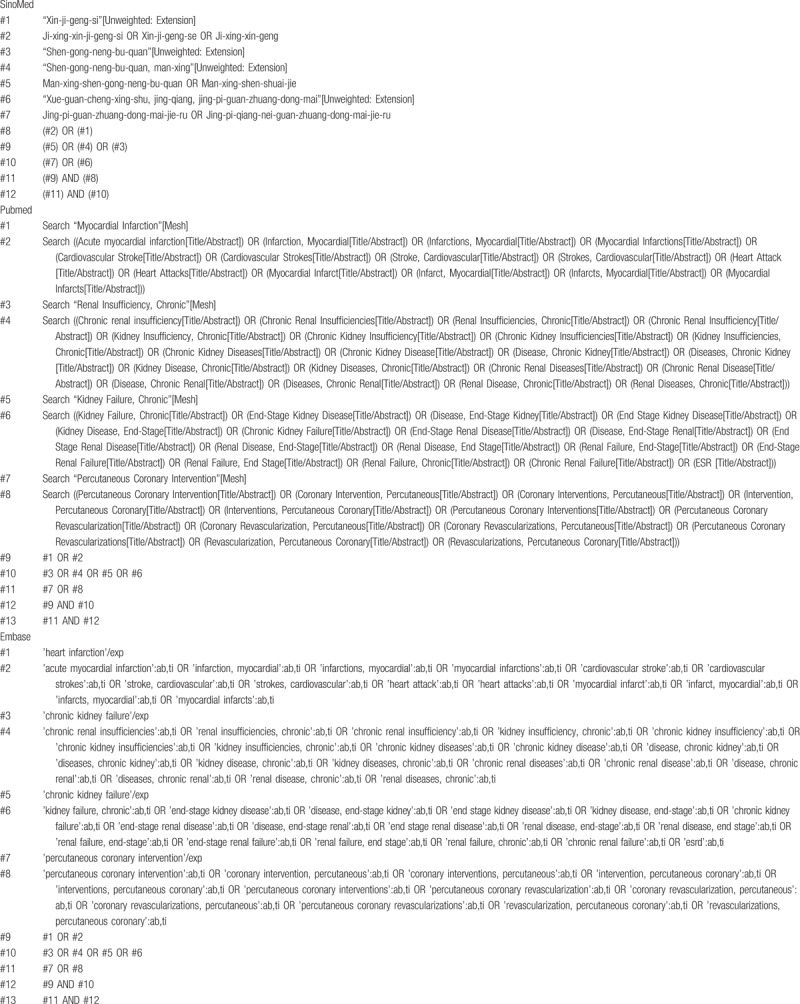
Retrieval strategy.

#### 2.4.2 Searching other resources

We will conduct supplementary searches, including of the references in the retrieved articles and supplementary searches of other research data, such as the WHO Clinical Trials Registration Platform (http://apps.who.int/trialsearch/), the China Clinical Trials Registration Platform (http://www.chictr.org), and the US Clinical Trials Registry (http://clinicaltrials.gov/), to retrieve ongoing or unpublished studies.

### 2.5 Data collection and analysis

First, according to the above inclusion and exclusion criteria, 2 researchers will independently screen and extract the literature.

#### 2.5.1 Literature screening

**Figure 1:**
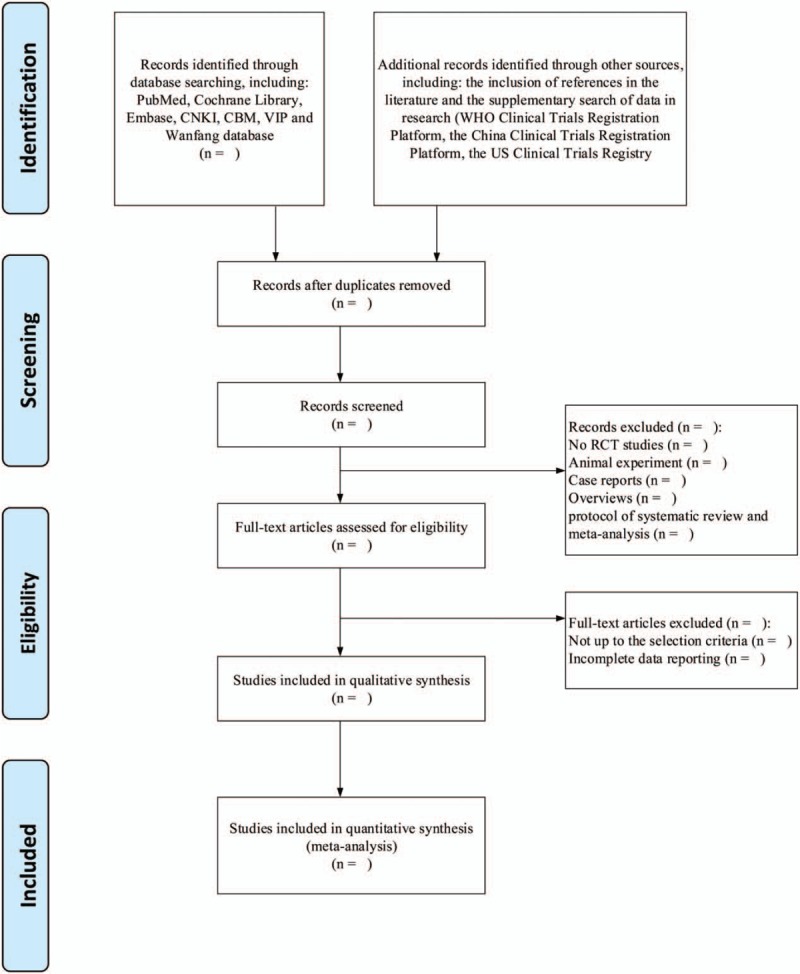
Flow diagram for study selection process used in this meta-analysis.

Two system evaluators will screen the literature, extract the data, and cross-check it after completion. In the case of disagreement, a third party will assist in judging whether to include an article. In the process of literature screening, we first will read the title and abstract of the article. After excluding the obviously unrelated literature, we will further read the full text to determine whether it is suitable for inclusion. A complete flow chart of the literature screen is shown in Figure 1.

#### 2.5.2 Data extraction

To avoid bias in the process of data extraction, the 2 researchers will independently extract data from the experiment using the same data recording form, and the differences between the extracted data will be resolved by negotiation and discussion. If necessary, some research data can be obtained directly from the corresponding authors. The following information will be collected from each study: authors’ information, year of publication, race (country), sample size, stage of disease, treatment regimen, clinical outcomes, and criteria for evaluating efficacy.

### 2.6 Study quality assessment

In this evaluation, case–control studies will be selected as the source of analysis, so the Newcastle–Ottawa Scale (NOS scale), which has been used by the Cochrane system, was selected for quality evaluation. The evaluation table includes the following 3 aspects^[18]^: the selection method of the case group and the control group, the comparison between the case group and the control group, and the evaluation of exposure. Under these 3 aspects, there are 8 items for case–control studies and cohort studies. It is easy to score the items that meet the requirements. Among them, the 4 items on the selected subjects count for 1 point each, the 1 item on intergroup comparability is worth 2 points, and the 3 items on exposure evaluation are worth 4 points combined, for a total of 10 points. Generally, studies with a score of >5 can be included in a meta-analysis.

### 2.7 Statistical analysis

#### 2.7.1 Measures of treatment effect

Enumeration data will be expressed by relative risk, and measurement data will be expressed by STD mean difference. Interval estimates will all be based on 95% confidence intervals (95% CI).

#### 2.7.2 Assessment of heterogeneity

Meta-analysis will be carried out using Stata 12.0 software. Statistical heterogeneity will be analyzed by the *I*^2^ test and *P* values. When *P* (≥.1) and *I*^2^ (≤30%) are considered to show no statistical heterogeneity among the studies, the fixed-effect model will be used to analyze data. If *P* < .1 or *I*^2^ > 30%, there is statistical heterogeneity among the studies, so we will trace the sources of heterogeneity and conduct subgroup analysis. If there is still significant heterogeneity, a random-effect model will be used to analyze the stability of the sensitivity analysis results. The publication bias will be analyzed by funnel plot and Egger test. *P* < .05 indicates statistical significance.

If there are missing data in an included study, they will be obtained by e-mail to the author of the study. If the author cannot be contacted successfully, the data will be obtained by statistical data conversion. If the data still cannot be obtained, the data will be excluded from this study.

#### 2.7.3 Subgroup analysis

Subgroup analysis is a grouping analysis of trials included in a study based on clinical characteristics of a specific clinical issue. In clinical research, sample size and the length of follow-up are the most commonly seen and make the results more pertinent. Subgroup analysis will be performed according to the number of lesion branches and the sample size of the experimental coronary arteries in the article.^[19]^

#### 2.7.4 Sensitivity analysis

Sensitivity analysis is used to analyze whether the results of a meta-analysis are influenced by human factors of the researchers, to explore the sources of heterogeneity, and to evaluate the stability of the meta-analysis results. Sensitivity analysis is mainly achieved by changing some factors. A random-effect model or fixed-effect model, relative risk or odds ratio, and mean difference or STD mean difference (SMD) analysis are used to compare whether the meta-analysis results have fundamentally changed after those changes. Studies can also be excluded one by one and the remaining studies submitted to meta-analysis to see whether the heterogeneity or the meta-analysis results fundamentally change. If sensitivity analysis shows a fundamental change in the heterogeneity or the meta-analysis results, then the stability of the meta-analysis results is poor. Care should be taken in the analysis of the results and the conclusions drawn from them.^[20]^

### 2.8 Publication bias

Publication bias is the most common systematic error in meta-analyses. Publication bias refers to the tendency that research results with statistical significance are more likely to be published than results with no statistical significance or invalid results, which leads researchers to obtain different probabilities of positive and negative results when consulting the literature, thus overestimating the true effect based on the comprehensive analysis of published research literature. In this study, funnel plots and Egger regression analysis will be used to evaluate publication bias. The funnel diagram can directly reflect whether the effect value of the original study is related to the sample size.^[21]^ If all the studies are symmetrically arranged around the centerline of the funnel graph, it shows that there is no publication bias; if not, there is publication bias. Funnel diagram interpretation is subjective to some extent. All the studies will be submitted to Egger regression analysis to calculate whether there is publication bias. If *P* > .05, this will show that there is no statistical significance in Egger test, that is, there is no obvious publication bias. When *P* ≤.05, there is publication bias.

## 3 Discussion

AMI is a serious clinical emergency with a rapid progression of illness and a high mortality rate, which seriously threatens the quality of life and health of patients.^[22]^ In recent years, its morbidity and mortality have been increasing. Most patients with AMI have CRI.^[23]^ AMI is a sudden interruption of coronary artery blood supply because of coronary artery disease, leading to myocardial ischemia and necrosis, cardiac function decline, and insufficient renal artery blood perfusion, leading to acute renal injury (ARI). Relevant studies have shown that the number and activation of macrophages in atherosclerotic plaques of patients with CRI increases significantly compared with those without renal impairment. With the deterioration of renal function, the expression of inflammatory cytokines (interleukin-6, tumor necrosis factor-alpha, among others) also show an increasing trend.^[24]^ Overseas studies suggest that the level of the serum myocardial enzyme CK-MB, brain natriuretic peptide level, hypertension, and diabetes mellitus are independent risk factors^[25]^ for AKI in patients with AMI.

PCI is recognized as the most effective method for the treatment of this disease. It is superior to urokinase intravenous thrombolysis in restoring myocardial reperfusion, reducing mortality, and improving prognosis. Although PCI can effectively relieve vascular stenosis and improve clinical symptoms, PCI itself can induce platelet aggregation through plaque rupture, which will lead to the formation of acute thrombosis and complications such as thrombosis, no reflux/slow flow, acute coronary occlusion, and even death. It is still a difficult and important problem to be solved urgently in the clinic, and some studies have shown that PCI can be an independent factor for renal injury after acute myocardial ischemia.^[26]^ Therefore, it is very important for the treatment and prognosis of patients with AMI and CRI to explore the clinical efficacy and safety of PCI.

We can see from the above that there are many studies on PCI in the treatment of AMI. At the same time, PCI has been widely used in the clinical treatment of AMI, and its efficacy is better than that of intravenous thrombolysis. However, there are no data on the clinical efficacy or safety of PCI in the treatment of AMI with CRI at present. Therefore, the purpose of this study is to provide medical evidence for the treatment of patients with AMI and CRI. However, certain factors existing in the systematic evaluation may cause potential publication bias: methodological differences, such as reagents, instruments, and thresholds; different lengths of time from chest pain to presentation at the hospital; differences in populations between studies, such as the proportion of men and women and patient age; different numbers of documents in Chinese and English, different numbers of people in different countries or regions, and regional differences in research methods.

## Author contributions

Shi-Hui Xiao and Liang Luo proposed the concept of this study and designed this systematic review. Shi-Hui Xiao registered the protocol of systematic review and meta-analysis. Liang Luo, Wen-Qing Xu, Ri-Xiang Zhong and Feng Chen are responsible for the collection, collation and statistical processing of the literature. All authors participated in the drafting of the first draft of the paper. Shi-Hui Xiao reviewed and proofread the paper. All authors agree to publish the paper publicly.

**Conceptualization:** Shi-Hui Xiao, Liang Luo

**Data curation:** Liang Luo, Wen-Qing Xu, Ri-Xiang Zhong, Feng Chen

**Methodology:** Liang Luo, Wen-Qing Xu, Ri-Xiang Zhong

**Software:** Ri-Xiang Zhong, Feng Chen

**Supervision:** Shi-Hui Xiao, Liang Luo

**Writing – original draft:** Liang Luo, Wen-Qing Xu, Ri-Xiang Zhong, Feng Chen, You-Lin Fu, Peng Zhang

**Writing – review & editing:** Shi-Hui Xiao.
